# Circulating Omentin Levels in Heart Failure: A Case–Control Study

**DOI:** 10.3390/ijms27093998

**Published:** 2026-04-29

**Authors:** Diego Currò, Edoardo Vergani, Maria Anna Nicolazzi, Angela Maria Rita Favuzzi, Flavia Angelini, Antonio Mancini, Andrea Silvestrini

**Affiliations:** 1 Dipartimento di Medicina e Chirurgia Traslazionale, Università Cattolica del Sacro Cuore, 00136 Rome, Italy; diego.curro@unicatt.it (D.C.); edoardo.vergani@outlook.it (E.V.); 2 Divisione di Medicina Interna e Malattie Cardiovascolari, Fondazione Policlinico Universitario “Agostino Gemelli” IRCCS, Università Cattolica del Sacro Cuore, 00136 Rome, Italy; 3 Dipartimento di Scienze della Salute e della Vita, Università Europea di Roma, 00163 Rome, Italy

**Keywords:** heart failure, HFpEF, HFrEF, omentin

## Abstract

Cardiovascular diseases remain the leading cause of global mortality, with heart failure (HF) representing a critical clinical endpoint. HF is traditionally classified into two distinct phenotypes based on left ventricular ejection fraction (LVEF): HF with reduced ejection fraction (HFrEF, LVEF < 40%) and HF with preserved ejection fraction (HFpEF, LVEF > 50%). While HFrEF is well-characterized and responsive to conventional pharmacological therapies, HFpEF remains therapeutically challenging due to its complex pathophysiology involving metabolic comorbidities and systemic inflammation. Emerging evidence suggests that adipokines may play a role in these inflammatory pathways. The present study aimed to evaluate and compare circulating levels of omentin, a recently discovered visceral adipose tissue-derived adipokine, in patients with HFrEF and HFpEF. This study reports that omentin levels were significantly lower in both HFpEF and HFrEF compared to controls. Moreover, omentin is inversely correlated to BMI and significantly lower in HF subjects with COPD than in those with no medical history of COPD. In conclusion, omentin may represent a potential biological signal in heart failure; however, further validation in broader populations is necessary to establish its clinical relevance.

## 1. Introduction

Despite significant therapeutic advances, cardiovascular diseases (CVDs) remain the leading cause of global mortality. Heart failure (HF) represents a particularly prevalent, debilitating, and costly endpoint within the natural history of various cardiovascular conditions. In clinical practice, a fundamental distinction is made between heart failure with reduced ejection fraction (HFrEF) and heart failure with preserved ejection fraction (HFpEF), both of which have traditionally been classified based on left ventricular ejection fraction (LVEF). Specifically, HFrEF is characterized by an LVEF of less than 40% and is typically associated with systolic dysfunction, characterized by impaired cardiac contractility and blood ejection [1]. Conversely, HFpEF is defined by an LVEF of more than 50%, where the myocardium predominantly exhibits impaired relaxation and filling despite preserving systolic function [1].

On a clinical level, HFrEF patients typically present with a dilated left ventricle and thinned myocardial walls, structural changes frequently observed in ischemic heart disease or dilated cardiomyopathy. Functionally, this subtype is defined by impaired contractility. In contrast, HFpEF is commonly associated with concentric left ventricular hypertrophy, which contributes to increased myocardial stiffness. These structural attributes are usually secondary to chronic hypertension, aging, or metabolic comorbidities, including obesity and diabetes [2]. From an epidemiological perspective, HFpEF more frequently affects older adults and women, whereas HFrEF is more commonly diagnosed in younger male patients.

From a therapeutic standpoint, robust evidence supports the use of pharmacologic interventions—such as ACE inhibitors, beta-blockers, mineralocorticoid receptor antagonists, and angiotensin receptor–neprilysin inhibitors—in HFrEF, all of which significantly reduce hospitalization and mortality rates [1]. However, HFpEF remains challenging to treat, with these conventional therapies demonstrating limited success in improving clinical outcomes. Consequently, HFpEF management focuses primarily on symptom relief, blood pressure control, and comorbidity management. Although HFrEF and HFpEF arise from different underlying etiologies, both lead to significant morbidity, frequent hospitalizations, and a reduced quality of life [2].

Debate persists regarding whether these two phenotypes represent distinct points on a continuous spectrum or are entirely separate clinical entities. Our previous research demonstrated that circulating plasma irisin levels and antioxidant systems differ significantly between the two subtypes [3,4]. Building upon these findings, the present study investigates omentin (also known as intelectin-1), a recently discovered adipokine primarily secreted by the stromal-vascular fraction of visceral adipose tissue, specifically within the omentum [5]. The discovery of omentin shifted the understanding of how adipose tissue signals influence systemic inflammation and opened new perspectives in the study of metabolic pathways in cardiovascular diseases [6]. However, its specific role in heart failure phenotypes remains to be fully elucidated. Considering the distinct pathophysiological backgrounds of HFrEF and HFpEF previously discussed [1,2], we hypothesized that omentin levels might reflect these underlying differences.

The aim of the present study was twofold: first, to evaluate circulating omentin levels in patients with heart failure compared to healthy controls; and second, to explore whether omentin levels differed between HF phenotypes (i.e., HFpEF and HFrEF).

## 2. Results

The demographic and clinical characteristics, including comorbidities, of the study cohorts are summarized in Table 1.

**Figure 1:**
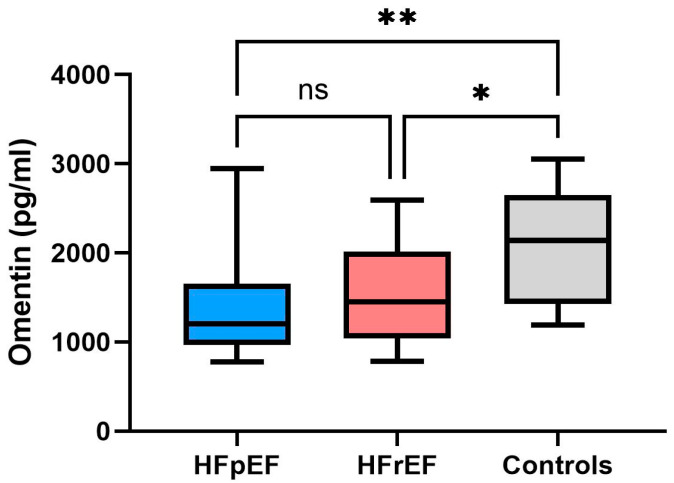
Comparison of plasma omentin levels between groups. The figure shows boxplots representing median, interquartile range and whiskers of the plasma levels of omentin (pg/mL) in the three groups: HFpEF, HFrEF, and controls. HFpEF: heart failure with preserved ejection fraction; HFrEF: heart failure with reduced ejection fraction. * *p* < 0.05 and ** *p* < 0.01; ns = not significant. The horizontal line within the box represents the median. The boundaries of the box represent the first and third quartiles.

As illustrated in Figure 1, plasma omentin levels were significantly lower in patients with both HF subtypes compared to controls. Instead, no significant differences in plasma omentin levels were observed between the HFpEF and HFrEF groups.

**Figure 2:**
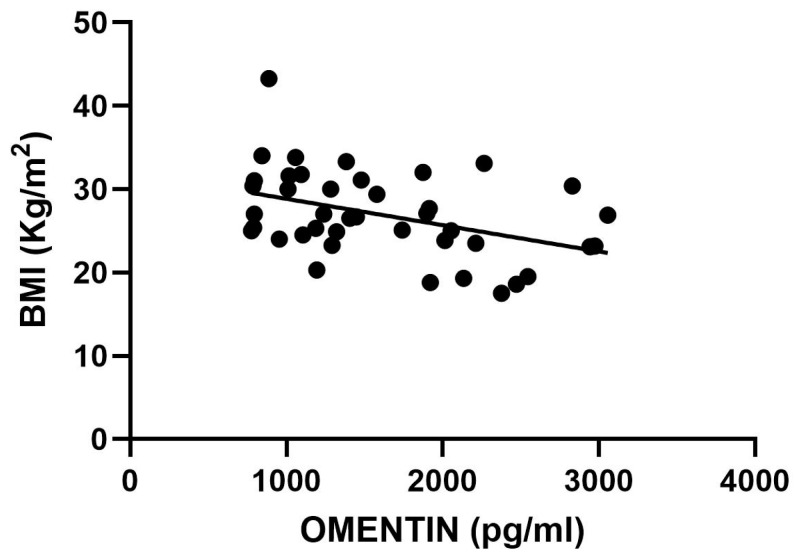
Plasma omentin and BMI correlation. Omentin levels (pg/mL) were inversely correlated with BMI (kg/m^2^) in the overall HF population.

Plasma omentin levels showed a significant negative correlation with body mass index (BMI) in the general HF population (see Figure 2).

To determine whether the observed differences in omentin levels between patients with heart failure (HF) and controls were influenced by body mass index (BMI), a logistic regression analysis was performed (see Table 2). In the unadjusted model, plasma omentin levels demonstrated a significant inverse association with the presence of HF (*p* = 0.005). After adjusting for BMI, this association remained statistically significant and maintained the same direction (*p* = 0.034). Therefore, a statistically significant but modest inverse association between omentin levels and the presence of heart failure was observed after adjustment for BMI (OR 0.999, 95% CI 0.998–1.000, *p* = 0.034). Table 2 shows logistic regression analysis outputs.

To account for the age imbalance between cohorts, a sensitivity analysis including age was performed. The inverse association between omentin levels and heart failure remained statistically significant, suggesting that the observed relationship is not entirely explained by age differences (OR 0.997, 95% CI 0.994–0.999, *p* = 0.013).

**Figure 3:**
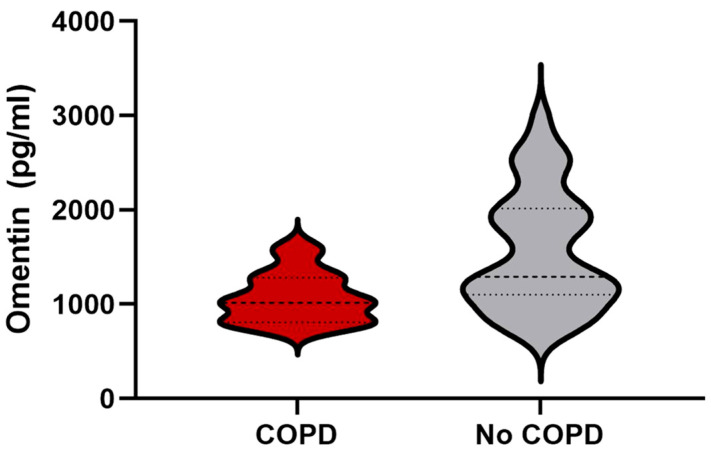
Plasma omentin levels and COPD. Here is shown the distribution of omentin values using a violin plot with the corresponding central tendency indicated. In patients affected by HF, plasma omentin levels were significantly lower in those with COPD than in those with no anamnesis of COPD. COPD: chronic obstructive pulmonary disease. Finally, in our cohorts, omentin levels were not significantly correlated with diabetes mellitus or peripheral arteriopathies.

Interestingly, chronic obstructive pulmonary disease (COPD) status was associated with lower circulating omentin levels. This result was observed both within the HF patient subgroups and across the entire study population, including healthy controls (Figure 3). These findings suggest that the association between omentin and COPD appears to be independent of the presence of heart failure.

## 3. Discussion

The present study aimed to evaluate possible differences in plasma omentin levels between HFrEF and HFpEF phenotypes, since in our previous reports, we found a different pattern for some parameters, such as plasma irisin and antioxidants [3,4].

Omentin is often reported as a promising adipokine peptide due to its beneficial impact on metabolism, and it has also been demonstrated to have anti-inflammatory properties [6]. It suppresses the expression of inflammatory cytokines, such as TNF-α, IL-6, and IL-1β, through inhibition of the nuclear factor kappa B (NF-κB) signalling pathways [7]. Omentin has also been found to modulate endothelial function, inhibit vascular smooth muscle cell proliferation, and foam cell formation, suggesting a potential cardiovascular protective effect [8].

Clinically, obese patients, those with type 2 diabetes mellitus (T2DM), metabolic syndrome, and atherosclerosis, have lower levels of circulating omentin. In contrast, higher omentin levels are usually associated with more favourable metabolic profiles, including increased insulin sensitivity, reduced systemic inflammation, and lower cardiovascular risk [6,9].

Emerging evidence also includes omentin in polycystic ovary syndrome (PCOS) [10], non-alcoholic fatty liver disease (NAFLD), and chronic kidney disease (CKD), whose levels are inversely correlated with the severity of these conditions. Moreover, omentin has been found to exert anti-proliferative and pro-apoptotic activity on certain cancer cell lines, such as colorectal and endometrial cancer, although its role in oncogenesis has not yet been explored [11].

With its broad spectrum of biological actions, omentin has also been proposed as a possible biomarker and therapeutic target for many metabolic, inflammatory, and cardiovascular disorders [12]. However, additional research is needed to elucidate the precise mechanisms underlying the regulation of omentin, its receptor interactions, and its long-term effects in vivo.

In relation to HF, some information on acute conditions has been reported: a high circulating concentration of omentin at discharge from hospitalisation could be related to a better prognosis [13]. Moreover, omentin values are associated with the severity of HF [12]. It has been proposed to consider serum omentin levels as the classification risk in HF for the stratification of patients [14]. The role of the omentin biomarker is highlighted in the HF with pre-existing coronary heart disease group [15]. In the elderly population in China with HFpEF, reduced omentin levels compared to controls have been described [16]. According to the multivariate logistic regression analysis, omentin emerged as an independent protective factor. Their results were also strengthened by ROC analysis, which showed a diagnostic efficacy similar to that of NT-proBNP [16]. No comparison was reported with HFrEF.

Given its pleiotropic activities, we investigated circulating omentin levels in patients with heart failure and explored its potential association with different clinical phenotypes. In our cohort, we found no significant differences between HFpEF and HFrEF, although both groups showed lower levels compared to controls. In agreement with the literature, we observed an inverse correlation between BMI and plasma omentin levels. COPD status was also associated with lower circulating omentin levels.

A possible explanation for the differences observed between irisin and omentin patterns could be related to their distinct tissue origins (myocytes and adipocytes, respectively), and potentially to their different roles in the modulation of oxidative stress [17].

Although irisin levels are elevated in HFpEF, possibly reflecting a compensatory response, they have been associated with myocardial damage in HFrEF. In contrast, the present observational study did not identify differences in omentin levels between heart failure phenotypes; these findings should be interpreted with caution and require confirmation in larger, well-matched cohorts.

The study has several limitations. First, the sample size is relatively small, which limits statistical power and the ability to control for multiple confounders. Second, the control group was younger than the HF population, reflecting the difficulty of identifying truly healthy older individuals without comorbidities; although a sensitivity analysis including age was performed and the association between omentin and heart failure remained significant, residual confounding cannot be excluded. Third, the observational and cross-sectional design does not allow any inference on causality or temporal relationships. These limitations may also contribute to the lack of association observed for T2DM and peripheral atherosclerosis in our cohort, in contrast with previous reports [18].

Heart failure with preserved ejection fraction (HFpEF) remains an increasing and incompletely understood clinical condition due to insufficient evidence. Generally, most epidemiological and clinical studies on HFpEF have used echocardiography for imaging purposes. Pathophysiology remains still not fully defined.

Overall, our findings suggest that circulating omentin levels are reduced in heart failure compared to controls, while no differences were observed between HF phenotypes. These results should be considered exploratory. Further prospective studies in larger, well-characterized populations are needed to clarify the potential role and clinical relevance of omentin in both heart failure models.

## 4. Materials and Methods

### 4.1. Study Population and Ethical Statement

This study was designed as an exploratory observational case–control analysis aimed at investigating potential associations between circulating omentin levels and clinically defined heart failure phenotypes, rather than establishing causal relationships. Thus, no prespecified hierarchical testing strategy was applied, the analyses were designed to identify potential associations between circulating omentin levels and heart failure phenotypes.

The study participants were recruited from the Department of Internal Medicine at the University Hospital “A. Gemelli”. The control group was selected among individuals undergoing clinical assessment who did not present any evidence of cardiovascular disease or relevant comorbidities. The control group was intentionally defined as a cohort of individuals without known comorbidities or ongoing pharmacological treatments, in order to minimize biological confounding factors affecting adipokine levels.

The research protocol was conducted in accordance with the Declaration of Helsinki (revised in 2013) and received formal approval from the Ethics Committee of the School of Medicine of Catholic University. All enrolled patients provided written informed consent before participation.

### 4.2. Clinical Diagnosis and Inclusion Criteria

The diagnosis of heart failure (HF) was independently verified by senior cardiologists based on comprehensive clinical history, physical examination, laboratory tests, and echocardiographic parameters, following the European Society of Cardiology (ESC) guidelines [19]. Patients were categorized into two groups based on the Left Ventricular Ejection Fraction (LVEF). HFrEF Group: Patients presenting with HF symptoms and an LVEF below 40%. HFpEF Group: Patients presenting with HF symptoms, an LVEF of at least 50%, and an NT-proBNP level exceeding 123 pg/mL. Additionally, the presence of at least one of the following was required: (a) significant structural heart disease (e.g., left ventricular hypertrophy or left atrial enlargement) or (b) diastolic dysfunction. The exclusion criteria included uncontrolled hypertension (blood pressure > 140/90 mmHg), alcoholism, drug abuse, liver dysfunction (transaminases > twice the normal upper limit), end-stage renal disease, malabsorption syndromes, and gastroesophageal reflux disease.

The control group consisted of 17 individuals, 32–57 years old, without significant comorbidity, and all the parameters tested were within the normal range. Twenty patients with HFrEF, aged 42 to 88 years, and 20 patients with HFpEF, aged 64 to 88 years, were recruited. The reduction in the HFrEF cohort from 20 to 18 subjects was due to technical outliers identified during the omentin assay. Specifically, these two samples yielded values outside the linear range of the standard curve (above the upper limit of quantification), rendering them analytically unreliable. Thus, to maintain data consistency and avoid the inclusion of extrapolated, unreliable values, these two outliers were excluded from the final statistical analysis. Therefore, 18 with HFrEF and 20 with HFpEF remained eligible. All participants were Caucasian and received treatment following the ESC of Cardiology guidelines with conventional therapy. As anticipated, comorbidities were more common among patients with HFpEF, with 45% having T2DM, 70% hypertension, 35% atrial fibrillation, 50% peripheral atherosclerosis, 50% non-end-stage chronic kidney disease, and 20% COPD, compared to patients with HFrEF, who had 28% T2DM, 89% hypertension, 56% atrial fibrillation, 39% peripheral atherosclerosis, 39% non-end-stage chronic kidney disease, and 22% COPD. The HF groups did not significantly differ in terms of age, sex, body mass, NYHA classification (all were class II or III), and physical activity levels, which were limited to sedentary activities.

### 4.3. Assays

Blood samples were collected between 8:30 and 9:00 a.m. after an overnight fast in a tube containing lithium heparin and centrifuged at 3000× *g* for 15 min at 4 °C. Plasma was stored at −80 °C until further use. Fasting glucose and insulin levels were measured using commercial kits with an ADVIA 2400 automatic analyser (Siemens, Erlangen, Germany). Serum NT-proBNP concentrations were determined using an electrochemiluminescence immunoassay on a Roche modular E170 analyser (Roche Diagnostics, Mannheim, Germany). The homeostatic model assessment (HOMA-IR) was used to measure insulin resistance, derived from fasting blood insulin (immunoreactive insulin: IRI, μUI/mL) and fasting blood sugar (FBS, mg/dL) levels, using the formula HOMA-IR = (IRI × FBS)/405. Plasma levels of circulating omentin were measured using a specific competitive enzyme immunoassay kit (catalogue number EIA-OME-1 from RayBiotech, Georgia, USA) following the manufacturer’s instructions. The assay was certified to have a sensitivity of 0.4 ng/mL and a detection range of 0.1–1000 ng/mL with intra-assay CV below 10% and inter-assay CV below 15%. Optical density was recorded at 450 nm using a microplate absorbance reader (iMark; Bio-Rad Laboratories Inc., Hercules, CA, USA) at a reading speed of 15 s. Each analysis was performed in duplicate.

### 4.4. Statistical Analysis

The primary analytical objective was to explore differences in circulating omentin levels between HF patients and controls, as well as between HF phenotypes, and to assess potential associations with selected clinical variables. No prespecified hypothesis-testing hierarchy was applied, and analyses should therefore be interpreted as exploratory.

No missing data were present for the primary variables analyzed.

The normality of quantitative data distribution was verified using the Shapiro–Wilk test and visual inspection of normality plots. Homogeneity of variances was evaluated through the Brown–Forsythe test. Depending on the data distribution, continuous variables were compared using one-way ANOVA, Welch ANOVA, or the Kruskal–Wallis test, as appropriate.

Qualitative variables were expressed as dichotomic (yes/no) and described using percentages. A *p*-value ≤ 0.05 was considered statistically significant for all analyses. Correlations between omentin levels and biochemical parameters were established using both linear and non-linear (semilogarithmic) regression models. Furthermore, a multiple logistic regression model was developed to evaluate the association between HF and omentin levels, adjusting for specified covariates, namely BMI since it was correlated with omentin in the HF population (Figure 2), and for subsequent sensitivity analysis. All statistical computations were performed using Prism GraphPad version 10.2.3 and JASP version 0.95.

## Figures and Tables

**Table 1:** General characteristics and comorbidities of the study cohorts. Median and interquartile range of Age, BMI, and NT-proBNP; % of clinical features (COPD, CKD, T2DM, peripheral arteriopathies, atrial fibrillation, hypertension). COPD: Chronic Obstructive Pulmonary Disease; CKD: Chronic Kidney Disease; T2DM: Type 2 Diabetes Mellitus. * *p* < 0.05 for HFpEF and HFrEF vs. Controls.

	**HFpEF** (*n* = 20)	**HFrEF** (*n* = 18)	**Controls** (*n* = 17)
**Gender**	Male: 12 (60%) Female: 8 (40%)	Male: 15 (83%) Female: 3 (17%)	Male: 5 (29%) Female: 12 (71%)
**Age** (years)	79 (73–83)	70 (60–77)	47.5 (32.2–57) *
**BMI** (kg/m^2^)	26.2 (24.6-31.9)	27.7 (23.9–31)	26.5 (21.8–28.7)
**NT-proBNP** (pg/mL)	2689.5 (863–4713.2)	3818.8 (1469.7–20,705.5)	-
**COPD**	5 (20%)	4 (22%)	-
**CKD**	10 (50%)	7 (39%)	-
**T2DM**	9 (45%)	5 (28%)	-
**Peripheral** **Arteriopathies**	10 (50%)	7 (39%)	-
**Atrial Fibrillation**	7 (35%)	10 (56%)	-
**Hypertension**	14 (70%)	16 (89%)	-

**Table 2:** Logistic Regression Analysis.

Independent Variable	OR (95% CI)	*p*-Value
Omentin	0.999 (0.998–1.000)	0.034
BMI	1.026 (0.874–1.206)	0.753

Outcome: presence of heart failure (yes/no). Model: adjusted for BMI, *n* = 55.

## Data Availability

The data presented in this study are available only on request from the corresponding authors due to the European Union General Data Protection Regulation (GDPR), to ensure the data privacy of the subjects participating in this clinical research project.

## Abbreviations

The following abbreviations are used in this manuscript:

HF	Heart failure
HFrEF	Heart failure with reduced ejection fraction
HFpEF	Heart failure with preserved ejection fraction
LVEF	Left ventricular ejection fraction
COPD	Chronic Obstructive Pulmonary Disease
